# Forearm Compartment Syndrome of a Newborn Associated with Extravasation of Contrast Agent

**DOI:** 10.1155/2013/638159

**Published:** 2013-06-09

**Authors:** Egemen Altan, Onur Tutar, Hakan Şenaran, Kerem Aydın, Mehmet Ali Acar, Levent Yalçın

**Affiliations:** ^1^Orthopaedics and Traumatology/Hand Surgery Department, Istanbul Bilim University, Florence Nightingale Hospital, 34000 Istanbul, Turkey; ^2^Radiology Department, Istanbul University, Cerrahpasa Medical Faculty, Istanbul, Turkey; ^3^Orthopaedics and Traumatology Department, Selcuk University Medical Faculty, Konya, Turkey

## Abstract

Extravasation of contrast agents is a possible complication of imaging studies. Although extravasations typically cause minimal swelling or erythema, they can lead to compartment syndrome when the volume of extravasation is high. In this article, we will present an exceptional case where an insignificant amount of contrast agent extravasation led to a forearm compartment syndrome in a newborn, who was treated with an extended fasciotomy. We would like to emphasize the preventive techniques and treatment options of this iatrogenic complication in newborns. Close followup of the patient by the nurses, awareness of the parents and the personnel in the radiology department are the most important preventive measures in this extremity-threatening complication. Forearm compartment syndrome due to contrast agent extravasation may progress more rapidly in newborns even with smaller amounts of extravasation and prompt recognition of the pathology and immediate intervention are unevitable.

## 1. Introduction

Neonatal compartment syndrome is a rare condition, which is typically caused by congenital anomalies, infections, difficult deliveries, and systemic abnormalities [1]. There are also reports in the literature that associate forearm compartment syndrome with extravasation of radiocontrast agents [2–4]. Forearm compartment syndrome may lead to functional disabilities, such as Volkmann's ischemic contracture and loss of neurological functions, if left untreated. Furthermore, an end-stage ischemic contracture may require salvage reconstructive operations, which typically have poor prognosis. 

In this case report, we will present an unusual neonatal forearm compartment syndrome that we attribute to extravasation of contrast agent from the peripheral venous catheter while performing a computed tomography (CT) scan. To our best knowledge, compartment syndrome in a newborn due to extravasation of a contrast agent has not been reported in the literature.

## 2. Case Report

A 23-day-old girl was referred to our clinic from the department of pediatrics with ecchymotic swelling of her left hand and forearm. This patient had been admitted to the department of pediatrics due to her pulmonary problems two days ago. Since she did not respond to the treatment, the patient underwent a contrast-enhanced computed tomography (CT) scan of the lungs. Approximately 10 mL of iohexol (Omnipaque, GE Healthcare) at a rate of 1.5 mL/sec was injected on the dorsum of the left hand. When the imaging study was completed—about 15 minutes after the contrast administration—the patient's mother noticed swelling in the neonate's hands. We think that the extravasation of the contrast agent occurred right after the contrast administration. In addition to swelling and tenderness of the hand, the neonate has also experienced visible discomfort and reduced mobility. In spite of conservative treatment including elevation of the upper extremit, and cold application, patient did not respond and the increased compartment did not resolve until surgical decompression. We did not perform local steroid injection in order to prevent any additional pressure increase.

**Figure 1:**
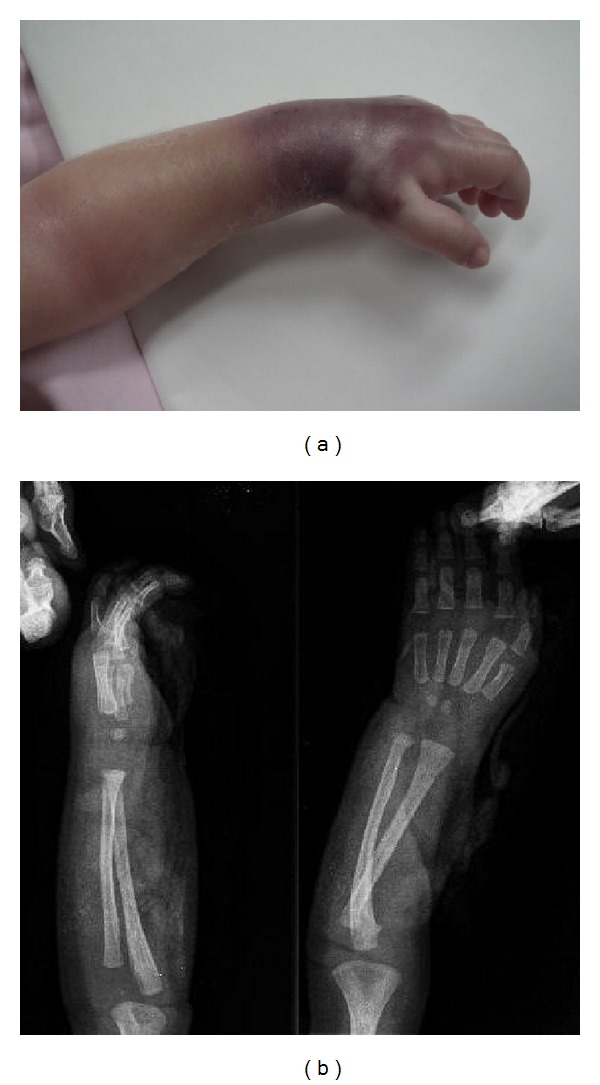
(a) Image showing the left hand and the forearm of the patient. Remarkable edema, swelling extending through the forearm can be observed. (b) X-ray of the patient shows a cloudy image indicating a considerable accumulation of contrast agent of both the forearm and the hand.

**Figure 2:**
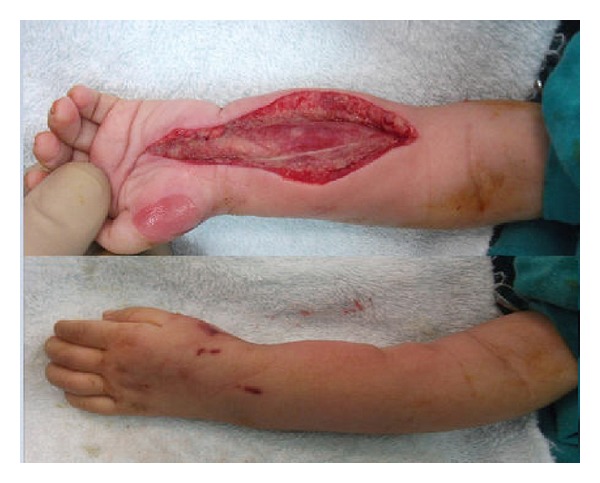
Intraoperative images of the volar fasciotomy and dramatic change of the color due to adequate vascularization of compression area.

**Figure 3:**
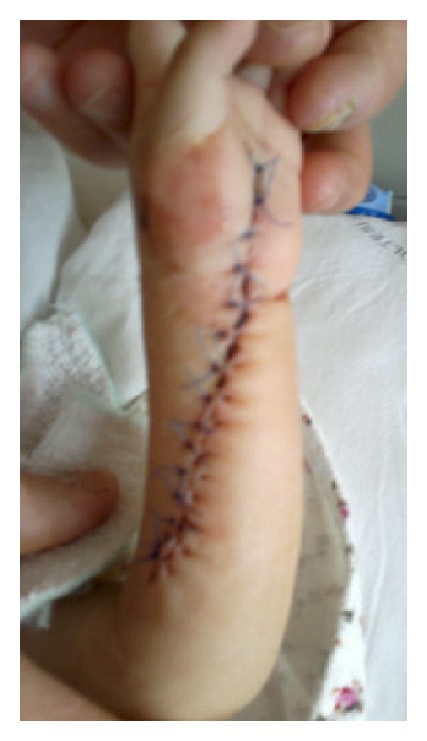
Postoperative view of the patient's forearm at the second week of surgery.

During physical examination, the patient's left hand and forearm exhibited marked oedema. A large and well-demarcated ecchymosis appeared on the dorsal side of the hand and a well-demarcated bullous skin area appeared on the thenar side. There was also a severe cyanosis extending to the forearm (Figure 1(a)). It was not possible to palpate the radial and ulnar pulses even with the Doppler ultrasonography. X-ray images were cloudy in the A-P and lateral views due to the contrast agent extravasation (Figure 1(b)). Although the pressure of the compartment was not measured, we clinically diagnosed the patient with compartment syndrome of the hand and forearm and immediately scheduled a volar fasciotomy including carpal tunnel, which was performed within two hours after extravasation. General anesthesia was not considered for the patient because of her advanced-stage pneumonia and an axillary block was performed. During the surgery, it was possible to observe the relief immediately after the fasciotomy incision and we noticed that the color and the consistency of the hand and the forearm have been improved. Moreover, overtension disappeared along the forearm and the dorsum of the hand. The postsurgical arterial Doppler ultrasound was positive for both the radial and ulnar arteries. Immediately after the surgery, color of the patient's skin turned to a pinkish hue. (Figure 2). On the day of surgery, we observed that the swelling was decreased dramatically and supple ROM of the fingers was noted. On the third day of surgery, X-ray images became gradually clearer. On the fourth day, we closed fasciotomy without the need of a skin graft and two weeks after the surgery, we removed the sutures (Figure 3). 

A year later, we were informed by the patient's pediatrician that the functional and neurological examinations during the followup did not reveal any abnormalities with free range of motion.

## 3. Discussion

Any condition that causes increased tissue pressure within a limited space can lead to compartment syndrome. Extravasation of contrast agent has been reported in the literature as a cause of hand or forearm compartment syndrome, and the immediate treatment of this condition has been highlighted [1–4]. This condition is extremely rare that Wang et al. reported only one subject suffered from compartment syndrome out of 475 patients who experienced extravasation of contrast agent on 69657 patients [5]. Most of the compartment syndrome cases in children have been attributed to various fractures and dislocations of the upper extremity. Compartment syndrome cases of the hand as a result of contrast agent extravasation have been reported in adults [2, 3]. However, neonatal forearm compartment syndrome due to ionic or non-ionic contrast medium extravasation has not been reported in the English literature. 

Even very small amounts of extravasation may lead to compartment syndrome in a newborn in a short time period. Furthermore, extravasation injuries are possibly more progressive conditions in newborns due to their more fragile vessel structure. The use of an automated power injector has been reported as another risk factor for unconstinous patients [6], and this may be the main reason for our neonatal patient's pathology. Despite the guidelines, there seems to be lack of great effort in the prevention of contrast extravasation at our institution, especially for those patients who have high risk for contrast extravasations. Therefore, we would like to emphasize the importance of close follow-up during application of automated power injectors especially for those newborns with an IV catheter.

It is not always possible for parents or caregivers to notice the changes in a neonate's behavior or health condition soon enough. In our case, although the CT examination took 15 minutes, the compartment syndrome occured in a very short time period. In a recent case report, even an elderly patient did not notify the nurses, despite the disturbing symptoms during CT scan [7, 8]. Thus, close monitoring of the patients—especially neonates—is essential to avoid these kind of extravasation injuries. Another recommendation includes application of nonionic contrast materials, which have a low osmolarity.

In contrary to previous reports [4, 7], clinical manifestations of compartment syndrome were settled within an hour due to the very young age of the patient. In these conditions, generally, treatment method is mainly based on the volume of fluid extravasation. However, in a newborn, clinical findings must be the most important factors for consideration of the pathology and management of the treatment. For cooperative older patients, elevating the limb and cooling the extremity with close followup can be useful for conservative management, which are recommended by most surgeons. However, it must be noted that wasting just a short time period with established clinical findings of a compartment syndrome can lead to irreversible changes and malpractice issues. The most important variables for development of compartment syndrome are the volume of extravasated contrast agent, the ionic or nonionic nature of the agent, vascular structure of the patients, and the age of the patient.

Involvement of both the hand and forearm led us to perform a volar fasciotomy instead of a partial dorsal web fasciotomy. There are reports of dorsal fasciotomies for isolated hand compartment syndromes, but clinical findings and negative results of the Doppler ultrasound along the forearm confirmed our decision of volar fasciotomy [9, 10]. Interpretation of the findings means that the vascularity of the whole forearm was corrupted and an isolated dorsal fasciotomy was not enough. However, after fasciotomy of the forearm, if the findings of the compartment syndrome do not relieve then an additional dorsal web fasciotomy should be mandatory. In such a case with the involvement of the forearm, one should not hesitate about a volar extended fasciotomy and an additional dorsal web fasciotomy must be kept in mind in order to save the extremity. It is mandatory to mention why a dorsal forearm fasciotomy was not done. It is common to perform a volar fasciotomy to release all the forearm compartments including carpal tunnel with only one incision.

In a recent report [7], contrast agent was shown on plain radiographs of the involved hand, and, similarly, we could demonstrate the extension of contrast agent extending to almost the elbow level which was also an evidence of a wide compartment syndrome. It is interesting to note that this compartment syndrome involved both the hand and the forearm, although the catheter was placed just on the dorsum of the hand. This denotes that the extravasation injuries may progress insidiously in a short time period in newborns.

Another point of interest is the anesthetic problems of this patient. She had serious pulmonary problems and in this emergency situation anesthesia team avoided from general anesthetics in order not to aggravate the pulmonary problems. Performing an axillary anesthesia was a necessity for our patient and requires even more experience for newborns. 

To our best knowledge, this was the first iatrogenic compartment syndrome reported in a newborn associated with extravasation of contrast agent and treated with an extended volar fasciotomy. Although rare, the risk of compartment syndrome should be considered in extravasations with very small volumes in a newborn. Close followup of the newborns and immediate intervention are the most important points to consider in this preventable pathology.
